# Preparedness of health care systems for Ebola outbreak response in Kasese and Rubirizi districts, Western Uganda

**DOI:** 10.1186/s12889-021-10273-2

**Published:** 2021-01-28

**Authors:** Michael Kibuule, Deogratias Sekimpi, Aggrey Agaba, Abdullah Ali Halage, Michael Jonga, Leonard Manirakiza, Catherine Kansiime, Dominic Travis, Katharine Pelican, Innocent B. Rwego

**Affiliations:** 1 School of Public Health, College of Health Sciences, Makerere University, P.O Box 7062, Kampala, Uganda; 2 Africa One Health University Network (AFROHUN), 16A Elizabeth Avenue, Kololo, Kampala, Uganda; 3 National Pharmacovigilance Centre, National Drug Authority, Ministry of Health, Kampala, Uganda; 4 One Health Division, College of Veterinary Medicine, University of Minnesota, St. Paul, MN USA; 5 Department of Ecosystems and Veterinary Public Health, College of Veterinary Medicine, Animal Resources and Biosecurity (COVAB), Makerere University, Kampala, Uganda

**Keywords:** Ebola, Disease outbreak preparedness, Health care systems, Infection prevention control

## Abstract

**Background:**

The level of preparedness of the health care workers, the health facility and the entire health system determines the magnitude of the impact of an Ebola Virus Disease (EVD) outbreak as demonstrated by the West African Ebola outbreak. The objective of the study was to assess preparedness of the health care facilities and identify appropriate preparedness measures for Ebola outbreak response in Kasese and Rubirizi districts in western Uganda.

**Methods:**

A cross sectional descriptive study was conducted by interviewing 189 health care workers using a structured questionnaire and visits to 22 health facilities to determine the level of health care system preparedness to EVD outbreak. District level infrastructure capabilities, existence of health facility logistics and supplies, and health care workers’ knowledge of EVD was assessed. EVD Preparedness was assessed on infrastructure and logistical capabilities and the level of knowledge of an individual health work about the etiology, control and prevention of EVD.

**Results:**

Twelve out of the 22 of the health facilities, especially health center III’s and IV’s, did not have a line budget to respond to EVD when there was a threat of EVD in a nearby country. The majority (*n* = 13) of the facilities did not have the following: case definition books, rapid response teams and/or committees, burial teams, and simulation drills. There were no personal protective equipment that could be used within 8 h in case of an EVD outbreak in fourteen of the 22 health facilities. All facilities did not have Viral Hemorrhagic Fever (VHF) incident management centers, isolation units, guidelines for burial, and one-meter distance between a health care worker and a patient during triage. Overall, 54% (*n* = 102) of health care workers (HCWs) did not know the incubation period of EVD. HCWs who had tertiary education (aOR = 5.79; CI = 1.79–18.70; *p* = 0.003), and were Christian (aOR = 10.47; CI = 1.94–56.4; *p* = 0.006) were more likely to know about the biology, incubation period, causes and prevention of EVD.

**Conclusions:**

Feedback on the level of preparedness for the rural districts helps inform strategies for building capacity of these health centers in terms of infrastructure, logistics and improving knowledge of health care workers.

**Supplementary Information:**

The online version contains supplementary material available at 10.1186/s12889-021-10273-2.

## Background

Ebola Viral Disease (EVD) outbreaks have occurred with increasing frequency in the last 15 years and the disease constitutes one of the biggest public health problems in Africa [1]. Many countries have reported EVD in the period between 1976 and 2018. Most notably is the Ebola outbreak in West Africa between 2014 and 2016 that affected mainly Liberia, Sierra Leone and Guinea [2]. Many frontline health care workers (HCWs) were affected due to lack of preparedness, poor and weak health care systems. In 2018, Democratic Republic of Congo (DRC) reported an EVD outbreak that appears to be still active [3]. Both the West African and DRC Ebola virus disease (EVD) outbreaks led countries globally to step up preparedness efforts. The World Health Organization (WHO) provided technical assistance to 14 priority countries to strengthen public health activities [1]. Furthermore, half (7/14) of the priority countries had achieved heightened level of preparedness according to WHO assessment criteria [1]. This WHO assessment checklist was developed for the EVD outbreak in West Africa in 2014 to look at factors such as proximity to highly affected countries, health systems developed and transport. In the wake of the DRC EVD outbreak of 2018, the World Health Organization Regional Office for Africa (WHO - AFRO) worked with nine neighboring countries including Uganda, to assess readiness and preparedness for EVD [4]. Uganda has since been tasked to scale up preparedness particularly border districts near North Kivu that experience frequent incursions of people from Eastern DRC (including Ituri province) into Uganda.

The first Ebola outbreak in northern Uganda of 2000 and 2001 is believed to have caught the country unawares with a naïve and inexperienced staff in response to contain the EVD outbreak. There were 425 cases with a case fatality rate of 53% [5]. In subsequent years, Uganda was in a position to quickly contain EVD outbreaks and minimize fatalities [5, 6] by putting in place incident command system, institute burial teams and multisectoral teams to manage the outbreak. This showed that the epidemic preparedness, response planning and training of multidisciplinary teams improved the country’s preparedness, alertness and response capabilities in controlling Ebola [7]. Other well managed outbreaks were Marburg disease around Rubirizi, Kamwenge and Kampala districts [8–10]. Both EVD and Marburg disease are priority zoonotic diseases in Uganda [11]. However, the low CFR for Marburg disease and the Bundibugyo Ebola virus disease outbreaks may have been due to their low virulence compared to the Zaire Ebola strain.

Yahaya et al. [4] reports that Uganda had made some progress on EVD in terms of coordination, having rapid response teams, Infections Prevention and Control (IPC), care management, dignified burials, and strengthening laboratory, epidemiological surveillance, risk communication and contact tracing. In addition, Uganda did have some level capacity building on EVD at the points of entry including having budgets and all logistics needed. However, details of preparedness for districts neighboring DRC are scarce. The country developed a Health Sector Strategic Plan (HSSIP III) for 2011–2015 that emphasized capacity building for response, early detection, prevention and control of newly emerging and endemic zoonotic diseases such as Ebola [12]. The level of preparedness for a disease outbreak determines the impact it has on the health care system and the individual health care workers. However, health care workers are known to lack knowledge of Ebola and other zoonotic diseases [13]. For instance, previous studies in Uganda found poorly prepared health care workers and non-adherence to the Universal Infection Prevention and Control precautions helped facilitate the rapid spread of Ebola during an outbreak. Starting in 2013, the United States Centers for Disease Control and Prevention (CDC) has supported the Ministry of Health in Uganda to establish an Emergency Operations Center (EOC) [14, 15]. Under the EOC, the Uganda Ministry of Health has followed the WHO Viral Hemorrhagic Fevers management guidelines and standard operating procedures (SOPs) as a means of ensuring preparedness for EVD and training health care workers [1].

Districts such as Kasese and Rubirizi, that are near the eastern border of the Ebola prone belt of DRC, are presumed to be high risk of EVD. The communities in these districts are poor and underserved and, as a result, still depend on hunting and consumption of game meat as source of livelihood. This increases their likelihood of interaction with wildlife and, therefore, the risk of contact with EVD reservoirs directly or indirectly [16, 17]. Uganda has porous borders whereby communities from neighboring countries travel easily to Uganda. The level of preparedness by districts near the border is not well known especially in terms of HCW knowledge of EVD, presence of infrastructure, logistics, rapid response teams, burial teams and simulations exercises to prepare health workers for an EBV outbreak. We set out to assess preparedness of the health care system and identify appropriate preparedness measures for Ebola outbreak response in Kasese and Rubirizi districts, Western Uganda.

## Methods

### Study area and setting

A cross-sectional study was carried out in health centers in Kasese and Rubirizi districts, in Western Uganda [18] from August to November 2016. Kasese district shares borders with the DRC with two administrative counties of Bukonzo and Busongora. About 26.5% of households are located within 5 km of the nearest Public Health Facility [19]. Uganda has guidelines for designating, establishing and upgrading different health care units ranging from Health Centre (HC) II to Hospital [20]. For instance, by virtue of their levels, the HC IV are by policy supposed to be equipped with logistics and supplies ready to identify, detect index cases and if possible, respond to Ebola and other deadly epidemic prone disease outbreaks. Kasese district is served by three hospitals, three HC IVs, 44 HC Level III, and 57 HC II [21]. The district has a total land area of 2724 km^2^ of which 885 km^2^ is reserved for Queen Elizabeth National Park and 652 km^2^ is reserved for Rwenzori Mountains National Park. The population density of Kasese District in 2014 was estimated to be 236 people per km^2^ [19]. On the other hand, Rubirizi district with a population density of 118 people per km^2^ [19] shares a border with Kasese and Bushenyi districts with two administrative counties of Bunyaruguru and Katerera counties. About 50% of Queen Elizabeth National Park lies in Rubirizi District. About 28% of the households are located within 5 km of the nearest Public Health Facility [19]. Rubirizi does not have a hospital whether Public or Private. However, it has one HC level IV, three HC Level III and 12 HC Level II facilities [21].

### Study design and variables

Study units composed of all the hospital and HC IV level facilities, and randomly selected HC level III facilities were established in both Kasese and Rubirizi districts [18]. Health Care Workers (HCWs), defined as any personnel working within a health unit such as medical doctors, medical clinical officers, nurses, laboratory technicians, midwives and nursing assistants, were recruited into the study through random selection within each health care facility. Other respondents recruited were district health management team members, in-charge of hospitals and health unit administrators. A total of 22 HC, including four hospitals, were assessed.

The sample size was estimated using Kish formular [22]. Assuming a 95% confidence interval and if the proportion of health care workers with knowledge on contact with body fluids of symptomatic person is 58.9% [23] and allowing alpha of 5% and non-response rate of 5%, approximately 391 health care workers were taken to be the number to be interviewed. However, only 187 HCWs were at the health facilities. These were interviewed using a questionnaire developed by the authors (S1). All hospitals and HC IVs in both districts were purposively selected because, by virtue of their levels, they are supposed to be equipped with logistics and supplies ready to respond to Ebola and other deadly epidemic prone disease outbreak. Fifty percent of the HC IIIs in each district were randomly selected using a ballot paper system, whereby a list of HC IIIs was obtained and a ballot paper with their names was written. The names were put on separate pieces of paper, thrown into a bag, shook and then randomly selected, one at a time, until the desired number was reached. From the selected health facilities, the names of health care workers were obtained from either the District Health Officers or the respective health facility administrators. Then the number of respondents was selected using simple random sampling. This was done using the computer-generated method whereby a list of names was generated into a column in a Microsoft Office (MS) Excel spreadsheet. Using the function = RAND the list of names and the random number were sorted by the random numbers.

In order to assess the level of knowledge on containment of EVD, names of HCW from the selected health facilities were obtained from either the District Health Office or the respective health facility administration. The names were then confirmed by fellow HCWs at facility level. Then the number of HCWs was selected using simple random sampling and proportionate to the number of HCWs in a given district.

### Independent and dependent variables

There were two categories of independent variables at 1) health facility level, and 2) individual HCW level. The health facility level variables considered were structural design of a health facility, district physical structural scale, and the catchment area /distance from facility to the vulnerable community. Regarding the structural design of the health facility, the district physical structural scale [24] was used to calculate the distances and spaces used for ascertaining safety precautions. A district health map was used to determine the distance or catchment area from each health care facility to the vulnerable community. The individual HCW variables included the respondent’s age, sex, type of employment, and job designation of the HCWs. The level of knowledge by a HCW was used as an dependent variable.

A WHO consolidated preparedness checklist developed for the West Africa Ebola disease outbreak of 2014 [1] was used to develop an adapted checklist for health facilities to measure the presence of: a VHF incident management center, a high-level isolation unit, clinical notification systems, a triage area spacious enough to ensure isolation of a patient in a holding area, and sufficient space to enable maintaining a meter distance among patients and between patients and HCWs. Data was also collected on the availability of protocols such as Infection Prevention and Control (IPC) guidelines, EVD management guidelines, burial and disposal guidelines, EVD treatment unit SOPs, policy guidelines/standard operating procedures, presence of surveillance systems, rapid response teams, Ebola and other deadly epidemic prone disease focal persons, burial teams, and table-top simulations and referrals [1]. Furthermore, the health facility logistics and supplies were measured to determine the capacity of a health facility based on the availability of the following: a health facility integrated disease surveillance and response check list, a standard case definition book for the 16 notifiable diseases, report forms for the 16 notifiable disease displayed, a budget, funds, lab and medical equipment, personal protective equipment (PPE), disinfectants & detergents, triple packaging kits, running water with soap and transportation of specimens. The knowledge of HCWs was measured based on the number of health care workers trained in Ebola and other deadly epidemic prone disease outbreak containment, the use of PPEs, infection control and practice, barrier nursing, quarantining, and triaging and isolation. The individual’s inclusion criteria were health care workers who have consented to the study, are working at health care facility of Health centre III and above and have worked in the district for the last 6 months.

### Data management

The data collected from health facilities and participants was cleaned, confirmed using different data sources such as asking more than one respondent about a given facility and checking for presence of a particular data by reading through documents. Data was then edited before being entered into Epi-data software. For any missing data, we went back to the field to collect it and/or verified from the hard copies. During the initial interview, we indicated to the facility in-charge, that we would follow up by phone in case we had follow up questions. Any other missing data from analysis was indicated so in the tables. The minimum completeness of data was 91% at facility level. The data was then analyzed using Stata version 14. EVD Preparedness was operationalized as the mean score of 5 domains: (a) two of the domains relate to Infrastructure and Logistical capabilities, and (b) three of the domains, measure the level of knowledge of an individual health worker about Ebola disease etiology, control and prevention. Infrastructure capability was assessed on a 14-item binary scale, where zero represented that there was no adequate infrastructure. The key infrastructural components were measured on a 14-item scale and if this gave a 14 “Yes” response, then that would demonstrate full availability of all the infrastructure for EVD outbreak and response. A score of equal to 7 or more was taken to be adequate. Logistical capacity was operationalized on a 23 item binary scale where zero shows no logistical capabilities and then 23 “Yes” responses demonstrated the maximum logistical capacity that was utilized in EVD detection, response and eventual management in case of the outbreak. Knowledge of Ebola etiology was measured on a 11 item binary scale, zero representing no knowledge and 12 points indicating a very high level of Ebola etiology knowledge. Knowledge on preventing EVD was elaborated on a 19-item binary scale point scale ranging from 0 to 19 and to this effect, if a health worker scored zero, then this demonstrated no knowledge of Ebola prevention measures. A 11-item binary scale was used to measure the knowledge of health care workers on Ebola control measures, zero indicating no knowledge on control measures whereas 11 points indicated that the HCW was very knowledgeable about the control measures.

Aggregation of these indicators into the five domains of the Ebola preparedness composite indicator was done to get an overall preparedness picture. The indicators were transformed into five domains by linear aggregation through summation. All the Yes responses were scored 1 indicating availability of a capability in a given domain and scored zero where respondents indicated absences of capability in a given domain. Mean score for each domain was calculated by summing up all scores in that domain and averaging them. The cut off for the binary outcome was set at the 50th percentile. Thus, a health facility that scored less than the 50th percentile was categorized as not prepared. The scale was highly reliable with an alpha score of 85% and 79% for infrastructure and logistical preparedness respectively. The level of health care knowledge was assessed with a cutoff set at 50th percentile. Health workers who scored less than the 50th percentile were categorized as having low levels of knowledge. The scale scored a Cronbach’s alpha reliability coefficient of 0.83.

At the univariate level, background characteristics of participants, infrastructure, logistic and knowledge indicators were summarized using descriptive statistics. For Health facility infrastructure and logistic capabilities, confounding analysis at bivariate level was carried by disaggregating the level by district and type of health facilities to explain the variance in the levels of preparedness. For the HCWs knowledge, bivariate analysis was carried out using contingency table analysis with level of knowledge as the dependent variable and the social demographic characteristics as the independent variable. The independent covariates namely, district, gender, age, education level, job description and religion, were regressed on the level of knowledge using the equation of a straight line in multivariate logistic regression. The unadjusted and adjusted odds ratio was used to assess the level of association at 0.05 significance level. A mixed-effect multilevel and fixed-effect binary logistic regression models were compared and the fixed effect binary logistic that had the smallest AIC value was used. A cut-off *p*-value for selection at bivariate level was set at 0.20 and forward selection method was used to select variables to include in multivariable logistic regression. The Area under the ROC curve was not fitted since we had more than one independent variables that were categorical in nature.

## Results

### Socio-demographic characteristics of the health workers

One hundred and eighty-seven (187) HCWs from both Kasese and Rubirizi districts participated in this study (Table 1). More than half of the participants in Kasese and Rubirizi districts were females (55.4 and 56.4% respectively). More than half of the HCWs in Kasese district (*n* = 75; 51%; Interquartile range [IQR] = 26–40) and in Rubirizi district (*n* = 21; 58.3%; IQR = 27–37) were in the 20–30 years age bracket. In both districts, the participants were inclined mainly to the Christian faith (Table 1). The highest level of education attained by the participants was a university degree. Nurses (enrolled and registered midwives) were the ones who mainly participated in this study (Table 1). However, majority of the health workers from both districts had attained tertiary level of education. Half of the HCWs in Kasese district (*n* = 74) and 82% from Rubirizi district (*n* = 32) were employed on a permanent basis by government (Table 1).

**Table 1:** Socio-demographic characteristics and distribution of Health Care Workers in Kasese and Rubirizi Districts, Uganda

Characteristic	Level	Kasese (*N* = 148) %(n)	Rubirizi (*N* = 39) %(n)
Gender			
	Female	55.4(82)	56.4(22)
	Male	44.6(66)	43.6(17)
Age (years)			
	20–30	50.7(75)	58.3(22)
	31–40	29.1(43)	25.6(10)
	41 and above	20.3(30)	17.9(7)
Religion			
	Catholic	29.3(43)	51.3(20)
	Muslim	4.1(6)	2.6(1)
	Pentecostal	0.0(0)	2.6(1)
	Pentecostal Baptist	2.7(4)	0.0.(0)
	Protestant	54.4(80)	28.2(11)
	Seventh-day Adventist	9.5(14)	15.4(6)
Education level			
	Primary level	1.4(2)	2.6(1)
	Secondary level	6.1(9)	2.6(1)
	Tertiary level	82.4(122)	82.1(32)
	University	10.1(15)	12.8(5)
Job designation			
	Senior Medical officer	0.0(0)	2.6(1)
	Medical officer	3.4(5)	0.0(0)
	Senior medical clinical officer	16.2(24)	10.3(4)
	Senior nursing officer	2.7(4)	2.6(1)
	Nurse	39.9(59)	56.4(22)
	Lab Technologist	20.9(31)	15.4(6)
	Other	16.9(25)	12.8(5)
Nature of employment			
	Permanent	50.0(74)	82.1(32)
	Temporary	43.9(65)	12.8(5)
	Volunteer	6.1(9)	2.6(1)
	Others	0.0(0)	2.6(1)

### Infrastructure capability and preparedness

Infrastructure level of preparedness for EVD outbreak and response was disaggregated by district. Three quarters of the health facilities in Rubirizi district had adequate EVD infrastructure and seven out of 18 health facilities in Kasese district had adequate EVD infrastructure. Preparedness in terms of infrastructure was also disaggregated based on the level of health facility. Nine out of ten of the HC IIIs in Kasese district were not prepared for Ebola Virus disease. All of the hospital level facilities in Kasese were prepared including 2 Clinics. In Rubirizi district, three out of the four health facilities, at levels HC III and IV were prepared.

Thirteen out of 22 of the Facility In-charges from both districts answered that the facilities did not have a copy of a case definition book. Twenty facilities visited did not conduct simulations, drills, and their Rapid Response Team (RRT) committees were non-functional (Table 2). Twenty of the facilities confirmed that they did not hold meetings. Health facility managers also confirmed that majority of members of the RRT committee had not been trained in Ebola preparedness and outbreak response. All Health Facility In-charges responded that they did not have burial teams. Fifteen health facilities did not have copies of SOPs for management of VHFs. Information on the quantity and location of Personal Protective Equipment (PPE) within 8 h of patient observation was lacking in some health facilities.

**Table 2:** Distribution of infrastructure capabilities among the 22 health centers in Western Uganda

Variable	Kasese (*N* = 18) % (n)	Rubirizi (*N* = 4) % (n)	Total N = % (N)
Are Ebola case definition books available?			
No	66.7(12)	25.0 (1)	59.1(13)
Yes	33.3(6)	7.05(3)	40.9(9)
Is the RRT/VHF committee in the facility functional?			
No	88.9(16)	100.0(4)	90.9(20)
Yes	11.1(2)	0.0(0)	9.1(2)
Is RRT/VHF committee constituted?			
No	61.1(11)	100.0 (4)	68.2(15)
Yes	38.9(7)	0.0(0)	31.8(7)
Does the RRT committee hold meetings?			
No	94.1(16)	100.0(4)	95.2(20)
Yes	5.9 (1)	0.0(0)	4.8(1)
How many HCWs have been trained in Ebola outbreak response?^a^			
None	47.1(8)	100.0(4)	57.1(12)
At least One	52.9(10)	0 (0)	0.0(0)
What is the number of PPE Active monitoring?			
None	52.9(10)	100.0(4)	61.9(13)
At least One	47.1 (8)	0.0(0)	38.1(8)
Do you have VHF focal persons?			
No	66.7(12)	100.0(4)	72.7(16)
Yes	33.3(6)	0.0(0)	27.3(6)
Do you have availability of information on quantity and location of PPE supplies within 8 h of patient under-observation?			
No	44.4(8)	100.0(4)	54.0(12)
Yes	55.6(10)	0.0(0)	45.5(8)
Is there a burial team in case of Ebola corpse?^a^			
No	100.0(4)	100.0(4)	100(4)
Are there any copies of policy guidelines and SOPs for VHFs?			
No	77.8(14)	25.0(1)	68.2(15)
Yes	22.2(4)	75.0(3)	31.8(7)
Is there a referral system in place?			
No	44.4(8)	0.0(0)	36.4(8)
Yes	55.6(10)	100.0(4)	63.6(14)
Does referral meet the recommended MOH requirements?^a^			
No	50.0(9)	75.0(3)	55.0(11)
Yes	50.0(9)	25.0(1)	45.0(9)
Do you conduct simulation and drills in preparation for EVD outbreaks?			
No	88.9(16)	100.0(4/4)	90.9(20)
Yes	11.1(2)	0.0(0/4)	9.1(2)

^a^missing data and/or responses

On a 14-points scale, it was observed that half of the health facilities had at least 50% of infrastructural and logistical capabilities. The cut-off point was a score of 7. Eleven out of the 22 of the Health Centre IIIs were observed not having adequate infrastructure and logistical capabilities. Out of the 22 health facilities, majority (*n* = 21) did not have VHF incident management centers nor did they have a high-level isolation unit (Table 3). There were no observed clinician notification files at almost a third (*n* = 7) of the health facilities. Nearly half of the health facilities did have a spacious triage area as well as spaces that could allow a one-meter distance between the HCW and the patient. Infection Prevention Control guidelines were only observed in only 17 health facilities. In addition, 12 facilities did not have EVD management protocols while 20 did not have treatment guidelines. All health facilities in both districts did not have burial and disposal of corpse’s guidelines. Personal Protective Equipment were not observed in 20 health facilities. It was observed that 15 health facilities had drugs, 18 had medical equipment and detergents, and running water was observed in the majority (*n* = 20) of the health facilities.

**Table 3:** Observed infrastructure and logistical capabilities at health facilities in Kasese and Rubirizi Districts

Variable	Kasese^a^ (*N* = 18) %(n)	Rubirizi^a^(*N* = 4) %(n)	Total (*N* = 22) %(n)
Is there presence of a VHF incident management centre? ^b^			
No	93.3(14)	100.0(4)	94.7(18)
Yes	6.7 (1)	0.0 (0)	5.3(1)
Is there presence of High-level isolation unit/biosafety level 4? ^b^			
No	100 (15)	100.0(4)	100.0(19)
Is there clinician notification? ^b^			
No	31.3(5)	25.0(1)	30.0(6)
Yes	68.8(11)	75.0(3)	70.0(14)
Is the triage area spacious? ^b^			
No	50.0 (8)	50.0(2)	50.0(10)
Yes	50.0(8)	50.0(2)	50.0(10)
Is space enough to enable requirement of keeping a meter distance from a patient? §			
No	43.8(7)	75.0(3)	50(10)
Yes	56.3(9)	25.0(1)	50(10)
Are there IPC guidelines? ^b^			
No	25.0(4)	25.0(1)	25.0(5)
Yes	75.0(12)	75.0(3)	75.0(15)
Are there EVD management guidelines and protocols? ^b^			
No	50.0(8)	75.0(3)	55.0(11)
Yes	50.0(8)	25.0(1)	45.0(9)
Are there protocols for burial and disposal of EVD corpses? ^b^			
No	100.0(16)	100.0(4)	100.0(20)
Are there Ebola treatment units SOPs? ^b^			
No	93.8(15)	75.0(3)	90.0(18)
Yes	6.2(1)	25.0(1)	10.0(2)
Is there laboratory equipment? ^b^			
No	33.3(5)	75.0(3)	42.1(8)
Yes	66.7 (10)	25.0(1)	57.9(11)
Are there PPEs? ^b^			
No	87.5(14)	100.0(4)	90.0(18)
Yes	12.5(2)	0.0(0)	10.0(2)
Are there enough drugs and vaccines? ^b^			
No	66.7(10)	75.0(3)	68.4(13)
Yes	33.3 (5)	25.0(1)	31.6(6)
Are there enough medical equipment? ^b^			
No	25.0(4)	0.0(0)	20.0(4)
Yes	75.0(12)	100(4)	80.0(16)
Do they have disinfectants and detergents? ^b^			
No	12.5(2)	0.0(0)	10.0(2)
Yes	87.5(14)	100.0(4)	90.0(18)
Is there running water with soap? ^b^			
No	12.5(2)	0.0(0)	10.0(2)
Yes	87.5(14)	100(4)	90.0 (18)

^a^Names of Districts; ^b^Missing data

### Logistical capability

Logistical preparedness of health facility is equivalent to having all the aspect falling within or above the cut off of 50th percentile on a 14-point scale. Twelve health facilities were not prepared in terms of logistics required for identification, detection and response of an EVD outbreak. All the four health facilities in Rubirizi district were not prepared compared to nine in Kasese. All of the four hospital level facilities in Kasese were prepared in terms of logistics. Four out of six of the HC IIIs in Kasese were not prepared in terms of logistics. All the three HC IIIs in Rubirizi district were not prepared logistically. The only one HC IV facility in Rubirizi district was also not prepared logistically. All the 22 health facilities that were assessed mentioned that they had never received any funds nor had a budget line to support EVD preparedness for identification of EVD index case and initial response (Table 4). In addition, 19 out of the 22 health facilities did not have laboratory equipment for collection of samples. Only one out of the 22 health facilities had the triple packaging of kits in store at the time of study. Half of the health facilities confirmed that they don’t have transportation mechanism for specimens to advanced laboratory for quick diagnosis. In both districts majority (*n* = 17) of the  health facilities did not have any PPE sets meant for protection in case of a suspect index case. Twenty health facilities reported that they had detergents and disinfectants (Table 4).

**Table 4:** Distribution of logistical preparedness of health facilities in Kasese and Rubirizi Districts

Variable	Kasese (*N* = 18) n (%)	Rubirizi (*N* = 4) n (%)	Total (*N* = 22) n (%)
Have you ever received a Budget/funds for EVD?			
No	100.0(18)	100.0(4)	100.0(22)
Do you have Lab equipment for collecting samples from EVD patients?			
No	83.3(15)	100.0(4)	86.4(19)
Yes	16.7(3)	0.0(0)	13.6(3)
How many triple packaging kits are in store in case of a suspected EVD patient?			
0	92.9(13)	100.0(4)	94.4(17)
1	7.1(1)	0.0(0)	5.6(1)
Do you have transport mechanism to send samples for advanced lab analysis?			
No	44.4(8)	75.0(3)	50.0(11)
Yes	56.65(10)	25.0(1)	50.0(11)
How many PPE sets do you have to aid infection prevention?			
0	81.3(13)	100.0(4)	85.0(17)
1^a^	18.9(3)	0.0(0)	15(3)
Does your facility have disinfections and detergents?			
No	5.6(2)	25.0(1)	9.1(2)
Yes	94.4(17)	75.0(3)	90.9(20)

^a^The health facilities with 1 to 3 Personal Protective Equipment (PPE) sets for infection prevention

### Knowledge and self-reported practices of EVD among the health care workers

One hundred and eighty-seven HCWs’ knowledge on preparedness was assessed and 85% (*n* = 160) mentioned that they knew what preparedness for EVD outbreak response is about (Table 5). Majority (59.4%) of the health care workers could not correctly answer the incubation period of Ebola. More than half (67.4%) reported that the there is no special triage for feverish patients. Majority (75.4%) measured the temperature of every patient. Fifty-six percent reported that the patients are asked about a set of symptoms. Only 52.9% reported the necessity to ask patients about a possible recent exposure to an EVD patient. Majority of the health care workers (59.9%) reported to have asked patients about travel history to EVD countries. Only 26.2% of the HCW reported doing full physical examination of patients. Nearly 53 % reported that they don’t draw blood for EVD testing. Majority of the HCWs (86.1%) did not know the infectivity period for EVD.

**Table 5:** Distribution of knowledge capabilities among Health care workers in Kasese and Rubirizi Districts, Uganda

Knowledge area (Variable)	Kasese (*N* = 148) %(n)	Rubirizi (*N* = 39) %(n)	Total (*N* = 187) %(n)
What is range of the incubation period of EVD?^b^			
Don’t Know	60.8(90)	53.8(21)	59.4(111)
Know	39.2(58)	46.2(18)	40.6(76)
Is there a need for Special triage area for feverish patients?^b^			
No	70.3(104)	56.4(22)	67.4(126)
Yes	29.7(44)	43.6(17)	32.6(61)
Do you measure temperature of every patient?^a^			
No	75.0(111)	76.9(30)	75.4(141)
Yes	25.0(37)	23.1(9)	24.6(46)
Is every incoming patient asked if he has fever?^a^			
No	65.5(97)	59.0(23)	64.2(120)
Yes	34.5(51)	41.0(16)	35.8(67)
Is every incoming patient asked about a set of symptoms?^a^			
No	43.8(64)	43.6(17)	43.8(83)
Yes	55.4(82)	56.4(22)	55.6(104)
Is every incoming patient asked about exposure to an EVD patient?^a^			
No	48.6(72)	1.0(16)	47.1(88)
Yes	51.4(76)	59.0(23)	52.9(99)
Is every incoming patient asked about travel to DRC and other EVD countries?^a^			
No	43.2(64)	28.2(11)	40.1(75)
Yes	56.8(84)	71(28)	59.9(112)
Is full physical examination conducted?^a^			
No	72.3(107)	79.5(31)	73.8(136)
Yes	27.7(41)	20.5(8)	26.2(49)
Do you draw blood for Ebola testing?^a^			
No	54.7(81)	46.2(18)	52.9(99)
Yes	45.6(67)	53.8(21)	47.1(88)
Do you take more precautions with suspected Ebola patients than with others?^a^			
No	86.2(128)	89.7(35)	87.2(164)
Yes	13.8(20)	10.3(4)	12.8(24)
Do you know the infectivity period for EVD?^b^			
No	85.1(126)	89.7(35)	86.1(161)
Yes	14.9(22)	10.3(4)	13.9(26)

^a^Self-reported practices; ^b^knowledge on different areas

**Fig. 1:**
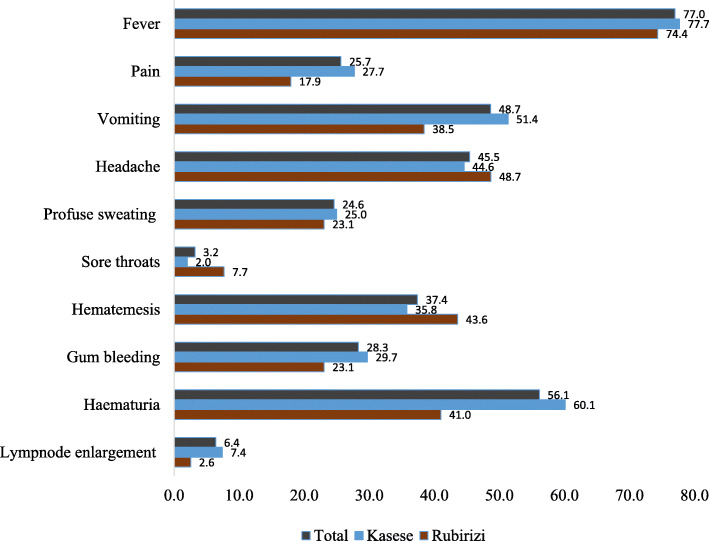
Health Care workers with knowledge on symptoms of Ebola. The question asked was “What are the common signs and symptoms seen in patients during early and late stages of EVD?” Most (77.7%) mentioned fever as the most sign of EVD

**Fig. 2:**
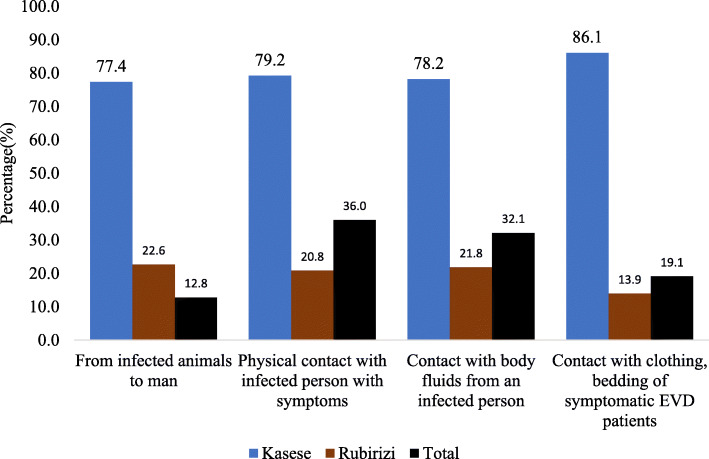
Health Care worker with correct knowledge on transmission of Ebola. The question asked was “How is Ebola transmitted?”

Most (77.0%) of the health care workers in Kasese and Rubirizi districts mentioned fever as the commonest sign of the Ebola Virus Disease followed by hematuria (56.1%), vomiting (48.7%), headache (45.5%) and hematemesis (37.4%) (Fig. 1). Less than half (36.0%; 68/187) of the respondents from Kasese and Rubirizi districts responded that physical contact with infected person is one of the key modes of transmission of EVD. Only 19.7% (37/187) of the health care workers from both districts responded that physical contact with body fluids from an infected person would transmit the disease. Less than half (32.1%; 59/187) of the respondents knew that contact with clothes and beddings of symptomatic EVD patients would be a mode of EVD transmission. In addition, very few HCWs knew that getting in contact with infected animals that are infected with the Ebola virus, would transmit the EVD to humans (Fig. 2).

Majority of the health workers (91.4%) reported to be wearing gloves and 76% used face masks as a standard precaution practice for infection prevention and control (Table 6). Only 49.25% of the health care workers knew and practiced hand washing before and after touching patients while only 31.6% of the HCWs knew that avoiding to recap needles after use was a standard precaution practice aimed at infection prevention and control.

**Table 6:** Infection, Precaution and Control (IPC) measures practiced by HCWs in Kasese and Rubirizi Districts, Uganda

Precaution	Kasese (*N* = 148) %(n)	Rubirizi (*N* = 39) %(n)	Total (*N* = 187) %(n)
Hand washing before and after touching patient	47.3(70)	56.4(22)	49.25(92)
Wearing gloves	91.2(135)	92.3(36)	91.4(171)
Use of face masks	72.3(107)	89.7(35)	75.9(142)
Use of goggles	44.6(66)	53.8(21)	46.5(87)
Avoid recapping of needles	30.4(45)	35.95(14)	31.6(59)
Use of sharp boxes	42.65(63)	46.2(18)	43.3(81)
Safe waste disposal	59.9(88)	64.1(25)	60.4(113)
Disinfection of equipment before use for another	45.3(67)	66.7(26)	49.7(93)

On an 11- points scale, more than half (54.5%) of the HCWs couldn’t respond to even half of the Knowledge capability questions. Rubirizi district appears 100% knowledgeable in a small aspect of logistics. Holding all other factors constant, the odds of being knowledgeable were not significant for gender, age, education level, job description and nature of employment among the different HCWs. In the adjusted model (Table 7), the highest education level was statistically significant. Health care workers who had attained tertiary education level were six times more likely to be knowledgeable compared to those with only secondary education (aOR = 5.79; CI = 1.79–18.70; *p* = 0.003).

**Table 7:** Knowledge of Health care workers on Ebola Virus Disease in Kasese and Rubirizi districts, Uganda

Independent variables^a^	Knowledgeable %(n)		COR (95% CI)	*p* - value	AOR (95% CI)	*p* - value
	Yes (*N* = 153)	No (*N* = 34)				
District						
Kasese	120 (78.4)	28 (82.3)	1.0	–	–	–
Rubirizi	33(21.6)	6(17.6)	1.51 (0.58–3.94)	0.39	–	–
Gender						
Female	80(52.3)	24(70.6)	1.0	–	1.0	–
Male	73(47.7)	10(29.4)	1.70(0.81–3.58)	0.160	1.41 (0.54–3.66)	0.482
Age						
41 and above	30(19.6)	7(20.6)	1.0	–	–	–
31–40	43(28.1)	10(29.4)	1.0(0.34–2.93)	0.995	–	–
20–30	80 (52.3)	17 (50.0)	0.84(0.33–2.19)	0.728	–	–
Education level^b^						
Secondary and below	6(3.9)	4 (21.1)	1.0	–	1.0	–
Tertiary level	143(95.8)	15(78.9)	6.36(2.05–19.66)	0.001^a^	5.79 (1.79–18.70)	0.003^a^
Job designation						
Nurse (Enrolled/Registered Midwife)	83(54.2)	24(70.6)	1.0	–	1.0	–
Clinician	40(26.1)	3(8.8)	3.13 (1.02–9.59)	0.046^a^	2.51(0.66–9.48)	0.176
Others	30 (19.6)	7 (20.6)	0.85(0.39–1.84)	0.697	0.78(0.27–2.29)	0.653
Nature of employment						
Temporary	64(41.8)	17(50.0)	1.0	–	–	–
Permanent	89(58.2)	17(50.0)	1.23(0.60–2.51)	0.573	–	–
Religion^b^						
Christian	146(98.0)	34(92.8)	1.0	–	1.0	–
Muslim	3(2.0)	4(10.5)	0.175 (0.02–0.48)	0.027^a^	0.096 (0.03–0.52)	0.006^a^

*95% CI* 95% Confidence interval; ^a^A cut-off p-value for selection at bivariate level was set at 0.20 and forward selection method was used to select variables to include in multivariable logistic regression; ^b^Missing data

## Discussion

### Infrastructure capability and preparedness

The study found that majority of the health facilities were not infrastructural prepared for EVD outbreaks. Most of the health facilities did not have case definition books and SOPs on EVD, and did not conduct simulation exercises or drills. In addition, most had non-functional Rapid Response Teams with minimal trainings. There was a general lack of VHF incident command centers and high-level isolation units in most facilities. Treatment and management guidelines for EVD were present in few facilities. In all facilities the burial and disposal guidelines were not readily available. On a positive note, most of the facilities had enough drugs, medical equipment, running water and detergents. However, personal protective equipment were limited in most facilities. These findings are particularly worrying, considering that western region of Uganda, especially in neighboring districts have previously reported outbreaks of VHF [9, 10, 25] and the fact there is a current EVD outbreak neighboring DRC [3]. Therefore, its worrying if these structures are missing in district since Ebola outbreaks normally cause panic and health care workers have been reported to abandon their posts. It is not a surprise to find deficiencies in infrastructure in decentralized health systems such as those in Uganda [26]. Loopholes in any of components of preparedness can increase the risk of transmission of EVD in health care and laboratory settings [27]. Despite such challenges Uganda has experience in management of VHFs such as Ebola and has set up systems to work on surveillance and coordination with neighboring countries such as DRC [28].

The findings of this study showed that lower health facilities such as HC III and IVs did not have most of the infrastructure. In a related study in Ghana, it showed that health facilities were not ready to handle EVD cases [29]. Limited funding from government to health systems to support equipping of these facilities to quickly identify, isolate and refer EVD cases may be the cause of such deficiencies. Small health facilities, such as HC II and III, especially those that have less than 200 beds are always less prepared compared to the bigger facilities with more bed capacity [30]. The current study found that hospitals were found to have the required infrastructure. Furthermore, at the district level it was found that Rubirizi district is more prepared to detect, identify and manage the index EVD case than Kasese District. This may be due to fact that Kasese district has a bigger population to serve, and thus more health facilities, than Rubirizi district. Rubirizi district is relatively newly created district new and does not have a district hospital established. Its highest health facility (HC IV) is currently serving as the district referral and thus will have almost the same level of funding as the Kasese District referral hospital.

Surprisingly, majority of the facilities did not have a copy of the case definition book despite fact that Uganda has availed this information to all health facilities [28]. Anecdotal evidence has suggested that even when these case definitions are available at health facilities, they are always not utilized and a copy will be kept in a records office. This supports the findings of this study which shows that the health in-charges may not even be aware of the availability of these materials at their stations. The case definition book of EVD forms part of the core infrastructures and lack of this book implies that health care workers are most likely to miss suspect cases and identification of potential cases of EVD. This poses not only a great risk to the heath care workers but also leads to straining of the health care system.

Majority of the facilities did not have RRTs and for those that formed the RRT committees they are reported non-functional. Therefore, meetings are not regularly held. This was confirmed by lack of evidence of existence of minutes of meetings. No wonder most health facilities reported that they didn’t conduct simulation exercises and drills in preparation for EVD outbreaks. In addition, majority of members of the RRT committees were not trained in Ebola preparedness and outbreak response. Ebola response requires harmonized coordination of all teams and the community. So, lack of rapid response teams means chaotic and improvised actions in case the case of Ebola emerged, and this will compromise communication and early containment of an outbreak. In Uganda, when there are epidemics RRT are constituted at district level. Currently, there are on-going efforts to train RRT [28] and conduct simulation exercises [31]. These are key in providing immediacy of experience that other training methods usually don’t provide [32].

Some of the health facilities assessed neither had VHF incident management centers nor high level isolation units. The study didn’t observe any clinician’s notification files at almost half of the health facilities. Almost half of the facilities assessed, had no space for triage as well as spaces that could allow a one meter distance between a HCW and a patient. Infection Prevention and Control (IPC) guidelines were only observed at a few of the health facilities. In addition, many of the health facilities didn’t have EVD management protocols and treatment guidelines for EVD. All the health facilities in both districts didn’t have guidelines for burial and/or disposal of corpses for EVD. This implies that if a suspected index case of EVD dies, there is no ready team in the nearby area to ensure safe and dignified burials of the corpse which puts the entire community at a greater risk of contracting the disease when they get involved in burying their people who may have died of EVD. WHO provides guidelines and checklist for countries to be better prepared for EVD at various levels [1]. Lack of isolation units led to some of health care workers being affected by EVD in Gulu, Uganda [6]. The local government should try its best to have isolation units in place especially for these districts that are near DRC where there is an on-going EVD outbreak.

Our study districts did not have burial teams for EVD. In a study conducted by Wamala et al. [33] early transmission of Ebola cases was due to burial rituals at community level. Last burial rites and practices in Africa involve family members preparing the corpse for burial including washing, touching and being in close proximity with corpse. This would be a risky practice if a family died from unsuspected Ebola disease. In the first EVD outbreak in northern Uganda, establishment of burial teams were found to be instrumental in containing the disease and stopping transmission within the community [6]. Safe and dignified burial is an important control measure in limiting transmission of Ebola at community level [34]. Mbonye et al. [28] recommended a national response team to train local burial teams be constituted since they form a critical infrastructural component. According to Okware [6], Uganda has started doing that.

### Logistical capability and preparedness

The study found that more than half of the health facilities were not logistically prepared. A related study health care logistics, the supply chain redesign of pharmaceutical products, and logistics system saturation is still an important challenge that the healthcare sector faces [35]. This implies that in case of a disease outbreak a lot health facilities will struggle to provide the required logistics to its staff and might have to depend on national level emergency mobilization efforts to respond adequately without putting themselves and other patients at risk of contracting infection. Patients infected with EVD, who seek emergency care, expose ‘front-line’ healthcare workers to significant risk of contracting the infection. Considering the highly contagious nature of the body fluids from individuals with symptomatic infection, dealing with Ebola mandates that healthcare workers follow standard safety precautions rigorously in order to safeguard themselves and the people with whom they interact [36].

In addition, the findings of this study show that Rubirizi district was doing poorly compared to Kasese in terms of logistics. The disaggregated data showed that all the facilities in Rubirizi didn’t have logistical abilities. This might be expected since these health facilities receive different quotas of funding from the Ministry of Health. The study also found that all health facilities, irrespective of level of health care, had no budget for responding to EVD nor did the facilities assessed have any funding towards VHF preparedness efforts. Primary Health Care funding (PHC) is very meagre and it is rarely used to handle any EVD preparedness activities. Decreased budgetary support from the government may be eroding the little gains of preparedness of Uganda’s health care system [37]. Funding for preparedness is key before, during and after an outbreak [38]. Resource mobilization at whatever stage of an outbreak, and especially preparedness, is vital because the fight against Ebola epidemics is highly resource intensive. This may be in form of medical and support staff, finances, vehicles, food, clothing, personal items or as hospital and laboratory equipment and supplies. To succeed in resource mobilization, there is need for multi-sectoral collaboration between ordinary citizens, civil society organizations, political and faith-based organizations, as well as local and international development partners and government departments [39].

Medical equipment was also another area that was missing in the facilities implying that, preparedness for, and response to the EVD index case may be routinely compromised. The medical equipment that were missing included containers for sample collection and storage, and PPEs. PPEs weren’t observed at health facilities implying that in case of the suspected index EVD case, even taking off the highly pathogenic specimen from the suspect, will either take long to be done, or the person taking it will have to remove the specimen without the PPE and that person will be more of a ransom to the EVD disease outbreak than a responder in actual sense. Presence of PPEs does not necessarily mean they will be used properly and in a timely manner by all HCWs. However, their presence will improve staff commitment and confidence while in isolation units [6]. The most available logistics in majority of health facilities were disinfectants and detergents, and transport for samples. This is to be expected as these are logistics that are used in other hospital activities on a day to day basis. For example, detergents are used in cleaning wards and disinfectants are used to clean surfaces soiled with blood and other fomites. This may explain their presence as the facilities will have a budget to purchase the items. Delivering of supplies and logistics in an epidemic situation, such as was in West Africa, has many challenges and countries need to learn the concept of prepositioning supply kits within the country as a way of preparedness [40].

### Self-reported practices and knowledge on etiology, transmission, control and prevention of EVD

In this study there was general low levels of self-reported practices and knowledge on etiology, mode of transmission, clinical signs and management of EVD in over 50% of the HCWs. This seems to agree with a study in Ethiopia amongst HCWs that showed nearly similar low levels of knowledge about EVD [41]. Annan et al. [29] also demonstrated the same in HCWs in Ghana during the period of Ebola outbreak in West Africa. In Nigeria, HCWs were found to have inadequate knowledge about EVD [42]. A similar study conducted by Benon et al. [18] in Kasese and Rubirizi districts showed total lack of knowledge about EVD amongst health workers. Interestingly, our current study found the self-reported level of knowledge of preparedness for EVD outbreaks slightly higher than as reported by Benon et al. [18]. Poor understanding of EVD among HCWs may put lives of people at risk [43].

The levels of wearing gloves and face masks as a standard precaution practice for infection prevention and control was very low. This may be due to general lack of PPEs as observed above. The HCWs who use face masks as a standard precaution practice for infection prevention and control was very low. They were very few HCWs who knew and appreciated that avoiding to recap needles after use on a patient is a standard precaution practice aimed at infection prevention and control. The health care workers who knew and practiced hand washing before and after touching a patient as a standard precaution for infection prevention and control was equally very low. Previous outbreaks of EVD in Uganda have shown incidences where HCWs are not using protective gear well or were taking the precautions put in place for granted [6]. Personal safety training focusing on safe wearing and removal of full-body equipment and working in pairs where colleagues watch over each other when wearing protective gears and when providing patient care were thought to be key in guaranteeing use of PPEs [39]. In the current study, very few HCWs knew and appreciated recapping of needles after use on a patient as a standard precaution practice aimed at IPC. 

A significant number of HCWs responded that physical contact is the commonest mode of transmission of EVD. Whereas this could be the commonest mode of EVD transmission, the other modes of EVD transmission were less mentioned. A case in point here was contact with body fluids from an infected person. This was less mentioned, yet it is equally a direct and obvious mode of transmission of EVD from person to the other. This simply means that the HCWs do not include contact with the body fluids of an EVD infected patient as a mode of transmission when designing health care messages while preparing to sensitize the public during their health talks. Very few HCWs knew that contact with clothes and beddings of symptomatic EVD patients would be an obvious and direct mode of EVD transmission. The implication on this is that precautionary measures are not taking place. The good news is that the level of knowledge seems to have improved or slightly better than in a previous study by Benon et al. [18] though comparison may not be made since the respondents may have been different. Very few of the health care workers knew that getting in contact with infected animals would transmit the EVD to man. The two districts border Queen Elizabeth national park where a case of Marburg was reported in a Bat cave [9] and Anthrax in hippos [44, 45]. Occurrences of such zoonotic diseases in the vicinity should encourage HCWs to learn more about diseases such as Ebola.

The current study has its limitations. For instance, recruitment of health care workers was based on names provided by the District Health officers. We addressed this limitation by confirming absence and/or presence of names of the HCWs at facility level. There were HCWs who could have had extra lessons of Ebola during their course of trainings. We defined whom to interview to avoid the selection bias and gave all the health care workers an even chance to participate by providing enough time for answering questions and asking us questions. The current study weighted all the indicators as the same yet in theory some components are more critical than others. Our indicators were based on the WHO Ebola preparedness checklist of 2014 [1]. The study design does not allow us to get a true picture of preparedness since it was conducted when the threat level for EVD outbreak in Uganda was at the minimum. During an epidemic situation some infrastructure and health facilities that may not receive funding when the threat level is low will be activated and funded. Countries tend to have more funding for case management and disease outbreak response in general than for preparedness [38]. Even though our results show that preparedness indicators can be used to monitor the extent of preparedness of communities, this tool seems to have been designed to be used mainly at national and sub-national level rather than community level. The preparedness indicator can be used as a tool to target interventions to the most vulnerable populations hence boosting preparedness. Countries can develop better tools that provide more detailed information on infrastructure and logistics needed for infectious disease outbreak preparedness such as EVD.

## Conclusions

Rubirizi and Kasese districts rated the same in terms of knowledge on EVD and preparedness in terms of logistics, such as laboratory equipment, budgets, PPEs and disinfectants. There was inadequate supply and preparation in terms of laboratory and medical equipment such as PPEs, triple packaging and special transportation mechanism. The knowledge of level of health care workers was slightly high as regards etiology while knowledge on EVD prevention and control was low. It is important that for a health system to be prepared all components such as knowledge of a disease, infrastructure and logistics should be in place. There is need to cascade preparedness and response efforts at global and national level to local community levels where disease outbreaks actually start from. District Rapid Response Teams should be constituted, trained, supported to hold regular meetings and conduct simulation drills. Absence of case definition books, burial teams and lack of dissemination of standard protocol like those on Infection Prevention and Control may put HCWs at risk of disease. The current study will provide a baseline of what is needed when it comes to preparing sub-national level health care systems, such as districts, in control and management of infectious diseases. Cross-border collaborations between Uganda and DRC is key to help in coordination of preparedness efforts as EVD spreads across borders during outbreaks.

## Supplementary Information

**Additional file 1.**

## Data Availability

The data used for analysis is all presented here.

## Abbreviations

DRC: Democratic Republic of Congo
EVD: Ebola Virus Disease
FGD: Focus Group Discussions
HC: Health Center
HCW: Health Care Workers
IPC: Infection, Prevention and Control
KII: Key Informant Interviews
MLHUD: Ministry of Lands, Housing and Urban Development
MOH: Ministry of Health
OHCEA: One Health Central and Eastern Africa
PPE: Personal Protective Equipment
RRT: Rapid Response Teams
SOPs: Standard Operating Procedures
UBOS: Uganda Bureau of Statistics
VHF: Viral Haemorrhagic Fever
WHO: World Health Organisation
