# Supplementary material for: Preparedness of health care systems for Ebola outbreak response in Kasese and Rubirizi districts, Western Uganda
Source: BMC Public Health. 2021 Jan 28;21:236. doi: 10.1186/s12889-021-10273-2 (PMC7844941; doi:10.1186/s12889-021-10273-2)
Supplement: Supplementary file 1 — Additional file 1. [file 12889_2021_10273_MOESM1_ESM.pdf]

Consent Form

Preparedness of Health Care Systems for Ebola Outbreak Response in Kasese and Rubirizi districts, Western Uganda

Introduction
I am …………. from Makerere University School of Public Health/OHCEA/Kasese District/Rubirizi District. I am conducting a study on "Preparedness of Health Care Systems for Ebola Outbreak Response in Kasese and Rubirizi districts, Western Uganda" to determine how prepared the health care workers and the health facilities are for Ebola Virus Disease. The data will inform the preparedness and response plans in case of Ebola outbreak in the districts mentioned.

You have been chosen to participate in this study as a health care worker. You will be asked questions to determine your knowledge about Ebola and practices regarding prevention of spread of Ebola. Participation in this study is voluntary. You can choose to participate or not to but I would be glad if you agreed. If you don't accept you won't be blamed at all and you can withdraw anytime without any risk to you or your family member. This interview will take approximately 15 minutes of your time. We envision not risks to you or your family if you participate in this study. Taking part in this study may not provide you with direct benefits. However, you or your community may benefit from the information collected here.

The information collected will be confidential. This information will be stored in a file that does not contain your name. I will use a code and data about you will be kept under key and lock and will not be accessed except by me and the study team and/or donors. You can withdraw at any time.

In case you have any questions or concerns, please contact Mr. Michael Kibuule on Phone: +256 772 949 430. For ethical issues, please contact the Makerere University School of Public Health Higher Degrees, Research and Ethics Committee at Phone No. +256-414-543872/543437/ 532207

Certificate of Consent to the study
I confirm that I have read the information sheet concerning study of preparedness of health care systems for Ebola outbreak response in Kasese and Rubirizi districts in western Uganda. I have had an opportunity to ask questions and these questions have been answered satisfactorily. I understand that my participation is voluntary, and I am free to withdraw my consent any time without giving any reason, without my legal rights being affected. I also understand that the data collected will be looked at by the OHCEA and Makerere University School of Public health research team representatives for purposes of determining how prepared the health care workers and health facilities are for Ebola Virus Disease outbreaks.
Name of participant…………………… Signature/Thumbprint………… Date (DD/MM/YY)……………..
Name of Witness ……………………….. Signature ……………………………. Date (DD/MM/YY)……………..
Name of Data collector………………… Signature …………………………… Date (DD/MM/YY)……………..

End of Consent form

Questionnaire

Date: ………../…………./………..
Form completed by
Name: ____________________ Position:_______________ District:_____________
Phone Number:________________________

Section A: Social-Demographic Characteristics

1. Serial Number:__________________
2. Location: Zone____________ Town _________________ Constituency: _____________
3. Gender Male ☐ Female ☐
4 How old are you? Insert your age in completed years ☐
5. What is your religion? ___________________
6. What is your education level? (*Choose one that applies*)
   a) Not educated
   b) Primary level
   c) Secondary level
   d) Tertiary level
   e) University Level
7. What is your job designation? (*Choose one that applies*)
   a) Senior Medical Officer/Consultant
   b) Medical officer
   c) Senior medical Clinical officer
   d) Senior Nursing officer
   e) Enrolled/registered Nurse/midwife
   e) Lab Technologist/Technician
   d) Other specify_______________
8. Nature of employment in this health facility? (*Choose one that applies*)
   a) Permanent
   (b) Locum/temporary
   (c) Volunteer
   (d) Others

Section B: Knowledge on Ebola

9. Do you know anything about Preparedness for Ebola outbreak response?
   a) Yes
   b) No
10. If yes, in (9) above, what does it means? (*Choose one that applies*)
   a) Observing the universal infection precautions and prevention protocols
   b) As in (b) above and having high level isolation unit and personal protective equipment (PPE).
   c) Performing a good burial to a corpse due to Ebola Viral Disease
   d) Others specify_________________
11. What is the incubation period of Ebola Viral Disease? (*Choose one that applies*)
   a) Few hours to 1 day
   b) 2 days to 3 weeks
   c) Greater than 3weeks
   d) I don't know

12. Of the following signs and symptoms, which one do you know is more specific to Ebola Virus Disease?
  a) Fever
  b) Muscles pains
  c) Vomiting
  d) Headache
  e) Profuse sweating
  f) Sore throats
  g) Hematemesis
  h) Gum bleeding
  i) Hematuria
  j) Lymph node enlargement ………………

13. How do you ensure that triaging by use of presenting complaints helps identify a potential suspected case of Ebola Virus Disease to confirm that the case is done to completion? (*Choose all that apply*)
  a) Special triage area for feverish patients is in place
  b) Measure temperature of every patient
  c) Every incoming patient is asked if he has fever
  d) Every incoming patient is asked about a set of symptoms (e.g., fever, diarrhea, vomiting, and sore throat etc.)
  e) Every incoming patient is asked about exposure to an Ebola Virus Disease patient
  f) Every incoming patient is asked about travel to Democratic Republic of Congo (DRC) and other Ebola Virus Disease endemic countries (Authors Note: *at time of study there was Ebola outbreak in neighboring country DRC)*
  g) Full physical examination
  h) Draw blood for Ebola testing
  i) Nothing more than the usual is done

14. When is Ebola Virus Disease regarded infective in terms of time periods (infectivity period)? (*Choose one that applies*)
  a) 0-2 days
  b) 3days before onset of symptoms
  c) 1st day of symptoms
  d) I don't know

15. What are the modes of transmission of Ebola Virus Disease? (*Choose all that apply*)
  a) From infected animals (non-human primate) to man
  b) By having direct physical contact with an infected person with symptoms
  c) By coming in contact with body fluids (e.g. saliva, urine, blood, stool etc.) from an infected person
  d) Person with symptoms of the Ebola Virus Disease
  e) By coming in contact with clothing, lining, bedding and materials of a symptomatic Ebola Virus Disease patients

16. How do you dispose the Ebola Virus Disease infected corpse? (*Choose one that applies*)
  a) Through cremation or burial in a sealed bag
  b) To the mortuary and the relatives pick the corpse for traditional burials
  c) The designated burial team joins the relatives for burial arrangement
  d) I don't know

Section C: Infection, Prevention and Control

17. Please tell me how you ensure standard precautions of infection prevention and control? (*Choose all that apply*)

a) Hand washing before and after touch a patient
b) Wearing gloves
c) Use of face masks
d) Use of goggles
e) Avoid recapping of needles
f) Use of sharp boxes
g) Safe waste disposal
h) Disinfection of patient care equipment before use for another patient
i) Creation of isolation units
j) Others.

17. How do you adopt the standard precaution practices and/or measures to prevent spread Ebola Virus Disease infection by the Health Care Workers after identifying a suspected case of Ebola Virus Disease? (*Choose all that apply*)

a) Isolation of the patient in holding area
b) Keeping a meter distance from patient
c) Observe universal precaution measures
d) Notify clinician
e) Obtain history of exposure or contact
f) Take temperature without touching patient
g) Physical examination
h) Wear mask, gloves
i) Wear full PPE
j) Notify Ebola Incident Centre
k) Refer patient
l) Good disposal of protective equipment

Thank you so much for your time.

End of interview
